# The incidence, impact, and risk factors for moderate to severe persistent pain after breast cancer surgery: a prospective cohort study

**DOI:** 10.1093/pm/pnad065

**Published:** 2023-05-15

**Authors:** Daniel L C Chiang, David A Rice, Nuala A Helsby, Andrew A Somogyi, Michal T Kluger

**Affiliations:** Department of Anaesthesiology, Perioperative & Pain Medicine, Waitemata District Health Board, Auckland, New Zealand; Department of Pharmacology and Clinical Pharmacology, Faculty of Medical and Health Science, University of Auckland, Auckland, New Zealand; Department of Anaesthesiology, Faculty of Medical and Health Science, University of Auckland, Auckland, New Zealand; Department of Anaesthesiology, Perioperative & Pain Medicine, Waitemata District Health Board, Auckland, New Zealand; Health and Rehabilitation Research Institute, School of Clinical Sciences, Auckland University of Technology, Auckland, New Zealand; Department of Molecular Medicine and Pathology, Faculty of Medical and Health Science, University of Auckland, Auckland, New Zealand; Discipline of Pharmacology, Faculty of Health and Medical Sciences, University of Adelaide, Adelaide, South Australia, Australia; Department of Anaesthesiology, Perioperative & Pain Medicine, Waitemata District Health Board, Auckland, New Zealand; Department of Anaesthesiology, Faculty of Medical and Health Science, University of Auckland, Auckland, New Zealand

**Keywords:** persistent pain, breast cancer, surgery, risk factors, impact, postmastectomy pain

## Abstract

**Background:**

Few Australasian studies have evaluated persistent pain after breast cancer surgery.

**Objective:**

To evaluate the incidence, impact, and risk factors of moderate to severe persistent pain after breast cancer surgery in a New Zealand cohort.

**Design:**

Prospective cohort study

**Methods:**

Consented patients were reviewed at 3 timepoints (preoperative, 2 weeks and 6 months postoperative). Pain incidence and interference, psychological distress and upper limb disability were assessed perioperatively. Clinical, demographic, psychological, cancer treatment-related variables, quantitative sensory testing, and patient genotype *(COMT, OPRM1, GCH1, ESR1,* and *KCNJ6)* were assessed as risk factors using multiple logistic regression.

**Results:**

Of the 173 patients recruited, 140 completed the 6-month follow-up. Overall, 15.0% (n = 21, 95% CI: 9.5%—22.0%) of patients reported moderate to severe persistent pain after breast cancer surgery with 42.9% (n = 9, 95% CI: 21.9%—66.0%) reporting likely neuropathic pain. Pain interference, upper limb dysfunction and psychological distress were significantly higher in patients with moderate to severe pain (*P* < .004). Moderate to severe preoperative pain (OR= 3.60, 95% CI: 1.13–11.44, *P* = .03), *COMT* rs6269 GA genotype (OR = 5.03, 95% CI: 1.49—17.04, *P* = .009) and psychological distress at postoperative day 14 (OR= 1.08, 95% CI: 1.02—1.16, *P* = .02) were identified as risk factors. Total intravenous anesthesia (OR= 0.31, 95% CI: 0.10 – 0.99, *P* = .048) was identified as protective.

**Conclusion:**

The incidence of moderate to severe persistent pain after breast cancer surgery is high with associated pain interference, physical disability, and psychological distress. Important modifiable risk factors were identified to reduce this important condition.

## Introduction

Breast cancer is the most diagnosed cancer in women worldwide.^1^ Approximately 80% of patients undergo surgical management which, when combined with adjuvant therapies, results in a 5-year survival rate of approximately 90%.^1^ Persistent pain after breast cancer surgery (PPBCS) is estimated to affect between 25% and 60% of patients, with approximately one in four reporting moderate to severe pain intensity.^2^^,^^3^ Given the volume of breast cancer surgery worldwide, the socioeconomic and healthcare burden of PPBCS is large due to the associated emotional distress, physical disability, upper limb disability and poorer quality of life.^2^^,^^4–6^

There are few studies on PPBCS from Australasia and thus the magnitude of the problem is poorly understood in this region^3–5^ as it is unclear how regional differences in ethnic composition, demographics, health care systems and inconsistent research methodology influence both incidence, impact and risk factors for PPBCS.^3^

As treatment of PPBCS is difficult, identification of at-risk patients is important to target management and prevention.^7^ Younger age, perioperative pain, axillary lymph node dissection, radiation therapy, education, lymphoedema, smoking, higher body mass index (BMI), hormone therapy, and chemotherapy have all been proposed as risk factors for PPBCS.^7–9^ Non-European ancestry, breast reconstruction, and axillary dissection in particular have been proposed as risk factors in a previous study involving New Zealand patients.^3^ Despite these, current risk prediction models can only explain approximately 30% of the variance in PPBCS incidence.^4^^,^^10^ To improve this, recent studies have included factors such as neurophysiological (assessed by preoperative quantitative sensory testing [QST]) and genetic variables with the more common patient and treatment-related factors in their risk factor analyses.^11–13^

Quantitative sensory testing (QST), as a biomarker of preoperative pain processing, has been proposed to identify patients at risk for both acute and persistent postsurgical pain (PPSP).^14–16^ Although there is a relationship between preoperative QST and acute postsurgical pain,^14^ the relationship with PPSP is less clear.^16^ Reported associations between PPSP and preoperative QST appear to be dependent on the surgical population studied and the QST measure; with conditioned pain modulation (CPM) and temporal summation (TS) of pain most frequently associated with PPSP.^16^ The role of preoperative QST to predict PPBCS has not been widely studied.^13^

Emerging evidence suggests that chronic pain per se has a heritable risk of approximately 45%^17^ and that genetic factors may partially explain individual differences in postsurgical pain.^18^ A consistent genetic basis for the development of PPBCS however has been elusive, often due to lack of replication in candidate genes studied.^18^ Nevertheless, several gene variants involved with neurotransmission (Catechol-O-methyl transferase [*COMT*]); voltage gated ion channel activity (Potassium channel gene, *KCNJ6*); neuroendocrine receptor interactions (opioid receptor, *OPRM1*), Tetrahydrofolate biosynthesis (GTP cyclohydrolase 1 [*GCH1*]); and inflammation (Interleukin 1 receptor type 2 [*IL1R2*]; Interleukin 10 [*IL10*]) have been consistently associated with the risk of developing PPBCS^12^^,^^19–22^ and PPSP.^18^ Furthermore, these gene variants may be subject to modulation through gene-gene interactions.^23^ To understand the individual variability in PPBCS development, recent studies have included exploratory assessments of gene variants with clinical, psychological and demographic factors as risk factors for PPBCS^13^ and PPSP^24^ development. Although not specifically powered as a genetic association study, these genetic factors (in an exploratory assessment) were incorporated with both treatment and patient factors into a comprehensive risk factor analysis to improve identification of at-risk patients, and to better understand the etiology of, and provide possible treatment targets for, PPBCS.

We hypothesize that the incidence of moderate to severe PPBCS in New Zealand is similar to other cohorts and is associated with high levels of pain interference, upper limb disability and psychological distress. Furthermore, key risk factors for the development of PPBCS may not only include clinical, demographic, and psychological variables, but may also include inherited factors and preoperative neurophysiology.

As such, this study had two aims: 1) to prospectively estimate the incidence and impact of moderate to severe PPBCS in a New Zealand cohort; 2) to identify key predictors for moderate to severe PPBCS development through assessment of clinical, demographic, and psychological variables as well as an exploratory, targeted assessment of inherited variation in genes previously associated with PPBCS (*COMT,*^19^*GCH1*^21^, *KCNJ6*^20^, and *OPRM1*^21^); chronic pain (*ESR1*^25^) and preoperative nociceptive processing (measured by QST).^14^

## Methods

This was a prospective cohort study of patients who underwent primary breast cancer surgery at North Shore Hospital and the Elective Surgery Centre administered by the Waitematā District Health Board (WDHB) in Auckland, New Zealand, between October 15, 2016, and August 15, 2020. New Zealand Health and Disabilities Ethics Committee (16/NTA/55/AM06) granted ethical approval to conduct the study.

Eligibility included 1) a diagnosis of breast cancer; 2) undergoing primary breast cancer surgery (mastectomy, partial mastectomy and lumpectomy); 3) >18 years old; and 4) being able to read and write English. Patients who had previous breast surgery, distant malignancy, or metastases were excluded from the study.

Consented patients were assessed at a preoperative clinic (within 14 days before the operation), and at two postoperative clinic appointments (2 weeks and 6 months after surgery). At each time point, patients were assessed for pain (at any anatomical site or pain in the breast, chest wall, shoulder and arm) incidence, pain quality and pain interference with activities of daily living, neuropathic pain, upper limb disability and psychological distress. Risk factors for developing PPBCS (clinical and demographic factors) were also collected at each time point. QST and blood collection for genetic assessment were performed before surgery. Data collected at each review are shown in Table 1.

**Table 1.** Outline of patient follow-up and data collection from before surgery to 6 months postoperative.

Category	Risk Factors			Outcome Measure ≥ 6 months After Surgery
	Preoperative	Intraoperative	2 weeks Postoperative	
Patient factors	Age, BMI, Ethnicity, Living with partner, Smoking status, highest education, medical history			Adjuvant Chemotherapy*, Radiation Therapy*, Endocrine Therapy*
Anesthetic and Surgical factors		Anesthetic Modality, Surgery type, Axillary Surgery, ICBN handling or transection		Repeat surgery at the primary surgical site*
Neurophysiology	QST (TS, PP40, CPM)			
Psychological distress	Psychological Distress (DASS21)		Psychological Distress (DASS21)	Psychological distress (DASS21)
Pain	Pain intensity at any site (BPI average pain Question 5), Likely neuropathic pain (DN4-interview)		Pain intensity in the breast/chest wall, axilla, shoulder, arm (BPI average pain Question 5), Likely neuropathic pain (DN4-interview)	Pain intensity in the breast/chest wall, axilla, shoulder, arm (BPI average pain Question 5), Pain treatment (BPI Question 7), Pain interference with ADL (BPI Question 9), Pain Quality (SF-MPQ-2), Likely neuropathic pain (DN4-interview)
Upper limb disability				Upper limb disability (DASH)
Genetic	Genotyping (*COMT, GCH1, KCNJ6, ESR1, OPRM1)*			

Variables collected as risk factors at 6 months denoted with *.

QST = Quantitative sensory testing; TS = Temporal summation; PP40 = Pressure pain 40; CPM = Conditioned pain modulation; DASS21 = Depression, Anxiety & Stress Scale 21; BPI = Brief Pain Inventory; DN4-interview = Douleur Neuropathique en 4 interview; SF-MPQ-2 = Short form Mcgill pain questionnaire 2; DASH = Disability of the arm, shoulder, and hand; BMI = Body mass index; ADL = activities of daily living.

### Anesthetic management

All patients received a simplified general anesthetic protocol which was utilized to limit variation in anesthetic practice and improve compliance. Details of this anesthetic protocol are provided in the Supplementary Material, Appendix 1.

### Predictor variables

Possible risk factors for developing PPBCS were based on findings from the existing literature^7–10^^,^^13^ and were collected intraoperatively and at 3 outpatient clinic appointments (preoperative, 2 weeks, and 6 months postoperative, Table 1).

#### Preoperative factors

Patient ethnicity, age in years (<50, 50–65, >65), ethnicity, BMI in kg/m^2^ (<25, 25–30, >30), living with a partner or living alone, smoking status (current, ex, never), highest educational level, and medical history (depression, anxiety, hypertension, diabetes mellitus, and chronic pain) were collected from the patient and medical record. Patient ethnicity was categorized by self-identified ethnicity as recommended by the New Zealand census. Due to their small sample size, those who belonged to the Māori, Pacific Island, Asian and Other ethnicity categories were pooled into the non-European ethnicity category.

The Short Form Brief Pain Inventory (BPI) and Douleur Neuropathique en 4-interview (DN4-interview) were completed to screen for preoperative pain intensity at any anatomical location, and likely neuropathic pain (DN4 interview ≥3/7), respectively. Pain intensity was categorized according to an 11-point numeric rating scale (0 = none, 10 = worst possible) of “average pain” from the BPI (question 5), where a score of 0–2/10 was considered no to mild pain, 3–10/10 moderate to severe pain and ≥1/10 any pain. The Depression, anxiety, and stress scale (DASS21) was used to screen for psychological distress.

##### Genetic Factors

Whole blood (8.5 mL) was collected into PAXgene blood DNA tubes (Qiagen, Hilden, Germany) and stored according to the manufacturer's instructions until required for extraction of genomic DNA (gDNA). The gDNA extraction was performed according to the manufacturer's instructions using the PAXgene Blood DNA Kit (Qiagen, Hilden, Germany). SNP genotyping was performed using the Sequenom^®^ MassARRAY iPlex platform (Sequenom^®^, San Diego, CA, USA). Details and protocols of the procedure are published elsewhere.^26^

Gene variants encoding catechol-o-methyl transferase enzyme (*COMT*), (rs6269, rs4633, rs4818, and rs4680),^19^ µ receptor (*OPRM1*), (rs1799971 and rs563649),^21^ guanosine-5-triphosphate cyclohydrolase (*GCH1*), (rs8007267, rs3783641, and rs10483639),^21^ estrogen receptor alpha (*ESR1*), (rs3020377, rs2234693 and rs9340799),^23^ potassium inwardly rectifying channel subfamily J member 6 (*KCNJ6*), (rs2835925, rs858003, and rs2835859)^20^ were selected for analysis based on published associations with PPBCS and chronic pain.

##### Preoperative Quantitative Sensory Testing (QST)

Quantitative sensory testing, including temporal summation (TS), pressure pain 40 (PP40) and conditioned pain modulation (CPM) were performed (methodology in Supplementary Material, Appendix 2) to identify patients with increased central sensitization or poorer CPM and who may be at increased risk of PPBCS.

#### Intraoperative factors

General anesthetic modality (volatile, total intravenous anesthesia), type of breast surgery (complete mastectomy, breast conserving surgery), surgical management of intercostobrachial nerve (not handled vs handled and not transected versus transected), axillary surgery (sentinel node biopsy, axillary node dissection, none), primary reconstruction with prostheses, or autologous flap at time of primary breast cancer surgery versus none were recorded.

#### Postoperative factors

Postoperative factors were collected at 2 time points (2 weeks and 6 months).

#### 2 Weeks postoperative

Pain intensity (no vs any; no to mild vs moderate to severe) in the breast/chest wall, axilla, shoulder or arm on the operative side was collected using the BPI (question 5, average pain). The DN4-interview was used to screen for likely neuropathic pain at these sites. The DASS21 was used to screen for psychological distress.

#### 6 Months postoperative

Data on adjuvant cancer therapies (adjuvant radiation therapy vs none; chemotherapy vs none; endocrine therapy vs none) and repeat surgery at the primary site of surgery (re-resection; breast conserving surgery converted to mastectomy or two-step Axillary lymph node dissection) were collected at 6 months after surgery.

### Outcomes and instruments

#### Primary outcome

The primary outcome was the presence of moderate to severe PPBCS, defined as average daily pain with a severity of NRS ≥ 3/10 (BPI question 5) in the ipsilateral breast, axilla, arm, shoulder or chest wall daily ≥6 months after surgery. A duration of ≥6 months was chosen to allow for the completion of adjuvant chemotherapy or radiotherapy and subsequent assessment of these variables as risk factors.^7–9^

Pain intensity was categorized according to an 11-point numeric rating scale of “average pain” from the BPI (question 5), where a score of 0–2/10 at ≥ 6 months after surgery was considered no to mild PPBCS, 3–10/10 moderate to severe PPBCS, and ≥1/10 any PPBCS. The primary outcome for the risk factor analysis was moderate to severe PPBCS in the ipsilateral breast/chest wall, shoulder, axilla, or arm (BPI average pain ≥3/10, ≥ 6 months after surgery)

#### Secondary outcomes

Pain interference with activities of daily living at 6 months after surgery were collected using the BPI (question 9). Details of analgesic medications usage and their effectiveness to treat pain at the time of 6-month BPI completion were also collected (BPI question 7).

Pain quality (continuous, intermittent, neuropathic and affective) and intensity ≥ 6 months after surgery were assessed using the Short Form McGill Pain Questionnaire 2 (SF-MPQ-2).^27^

The Douleur Neuropathique en 4-interview (DN4-interview) was used to screen for likely neuropathic pain ≥ 6 months after surgery. Patients with a total DN4-interview score of ≥3/7 were considered having likely neuropathic pain.^28^

Upper limb disability was assessed using the Disabilities of the Arm, Shoulder and Hand questionnaire (DASH) at 6 months after surgery. A difference of 10 points on the total DASH score was considered clinically important.^29^

The short version Depression, Anxiety and Stress Scale (DASS21) questionnaire was completed at 6 months after surgery to measure of overall psychological distress.^30^

### Statistical analyses

Depending on data distribution (normal vs non-normal), independent *t*-tests, one-way ANOVA, Kruskal-Wallis test, or Mann-Whitney *U* tests were used to examine between-group differences. The χ^2^ test or Fisher exact test was used to assess categorical variables. Confidence intervals were calculated using the modified Wald method.

All single-nucleotide polymorphisms (SNPs) were checked for agreement with minor allele frequency (MAF) reported in the literature, and χ^2^ analysis was used for violation of the Hardy-Weinberg equilibrium (HWE). The association between PPBCS and SNP was investigated using four different genetic models (additive, recessive, dominant and overdominant). Associations between PPBCS and the *COMT* haplotypes and diplotypes comprising rs6269, s4633, rs4818 and rs4680, respectively (GCGG, ATCA, and ACCG) were also assessed by combining and classifying patients with the GCGG/GCGG and GCGG/ATCA diplotypes as “group 1”; the ATCA/ATCA and GCGG/ACCG diplotypes as “group 2”; and the ACCG/ACCG and ACCG/ATCA as “group 3.”^24^

#### Logistic regression for identification of risk factors of moderate to severe PPBCS ≥ 6 months after surgery

Univariate logistic regression was performed to test the influence of variables for moderate to severe PPBCS. Subsequently, the two-stage linear setup procedure of Benjamini, Krieger, and Yekutieli to correct for multiple comparisons was performed.^31^ Multi-test adjusted *P* values smaller than 0.05 were considered statistically significant. Variables were selected for entry into multiple regression (full) model based on either statistically significant univariate association or previously published associations with PPBCS.^2^^,^^8^^,^^9^ A parsimonious final model was created using multiple logistic regression analysis by backward and forward elimination from the full model using stepwise elimination based on the likelihood ratio test followed by a manual selection. The AIC and BIC result were used to make a final judgement for final model. Multicollinearity was not considered problematic if factors had a variance inflation factor (VIF) < 5^32^. Sensitivity and specificity were calculated. Model goodness of fit and effect size were assessed using the Hosmer & Lemeshow and Nagelkerke *R*^2^ tests.^33^^,^^34^

A sample size of approximately 220 patients was required to estimate the incidence of PPBCS with a confidence limit of 5.5% on each side. Sample size calculation was based on a median prevalence of previous PPBCS estimates of 19.5%,^10^^,^^35^ and adjusted to account for a 10% loss to follow-up.^35^

A significance level of 5% (*P* < .05) and confidence intervals of 95% were used. Statistical analyses were performed using GraphPad Prism 8 for Windows (GraphPad Software, San Diego, CA, USA) or IBM SPSS Statistics for Windows, version 25.0 (IBM Corp, Armonk, NY, USA).

## Results

### Participants

**Figure 1.**
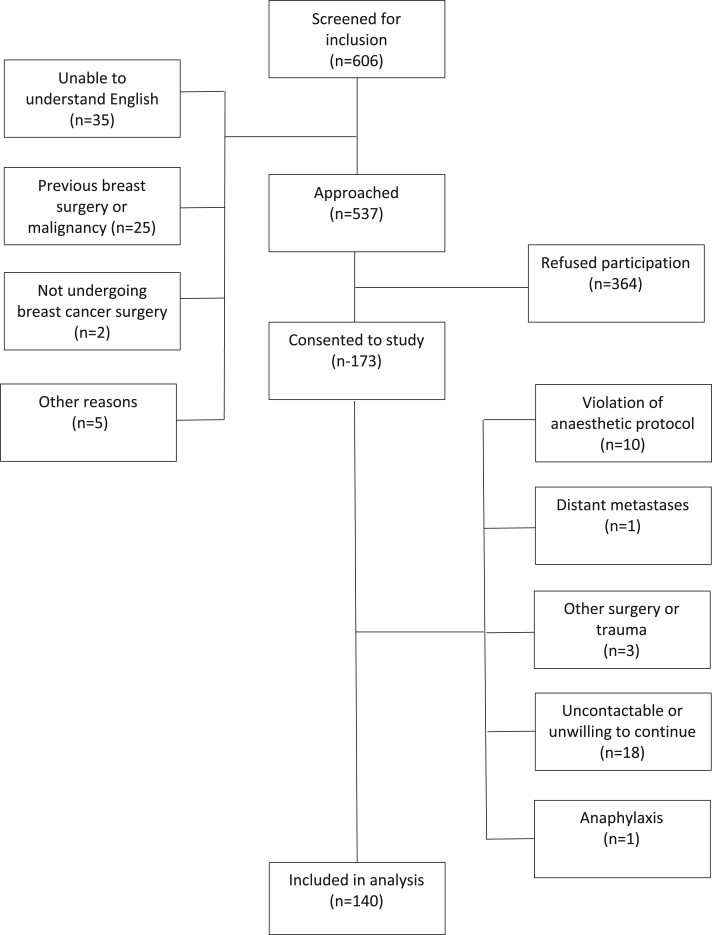
CONSORT diagram of patient recruitment.

A total of 173 patients were recruited, with 140 completing follow-up and included in the analysis. (Figure 1). The study was stopped early due to New Zealand COVID-19 restrictions, hence the recruitment target of 220 patients was not achieved.

Median (IQR) time from preoperative review to surgery was 7 days (IQR: 4 to 10 days); 88% of patients underwent surgery within 14 days of preoperative review. The median time from surgery to 2-week postoperative assessment was 15 days (IQR: 13 to 17 days), with PPBCS assessment at 6 month follow up a median of 6 months (IQR: 6–7 months). One patient did not present to the 2-week postoperative follow-up but attended the 6-month follow-up. No other data were missing from the 140 patients who completed the 6 month follow up.

Patient demographic and treatment variables were categorized according to PPBCS status (no to mild PPBCS vs moderate to severe PPBCS) and are displayed in Table 2. Preoperative pain, function and psychological assessments, postoperative pain scores and analgesic consumption according to PPBCS classification (no to mild vs moderate to severe) are displayed in Table 3. Demographic and treatment characteristics of patients included or excluded from the analysis are presented in Supplementary Table S1. Of note, the included and excluded patients differed with respect to mean age (57.8 years vs 62.5 years, *P* = .05) and postoperative adjuvant endocrine therapy (84.3% vs 60.6%, *P* = .01). There were no other significant differences between the two groups (all *P* > .05).

**Table 2.** Differences in baseline patient demographic, surgical and anesthetic characteristics, between patients who developed moderate to severe PPBCS and those who did not.

Patient Characteristics			No to Mild PPBCS (n = 119)	Moderate to Severe PPBCS (n = 21)	*P* value
Mean age (SD) at primary breast surgery; y			57.7 (11.7)	57.7 (13.6)	.99
Mean height (SD); cm			163.8 (6.6)	161.6 (6.4)	.16
Mean weight (SD) at primary breast surgery; kg			77.4 (16.6)	72.4 (21.5)	.23
Mean BMI (SD); kg/m^2^			28.8 (5.9)	27.6 (7.6)	.41
Ethnicity	European		93 (78.2)	15 (71.4)	.536
	Non-European		26 (21.9)	6 (28.6)	
		Māori	7 (5.9)	1 (4.8)	
		Pacific Island	7 (5.9)	0 (0.0)	
		Asian	10 (8.4)	5 (23.8)	
		Other	2 (1.7)	0 (0.0)	
Living with partner			79 (66.4)	13 (61.9)	.80
Highest education	Primary/Secondary		57 (47.9)	9 (42.9)	.39
	Tertiary undergraduate		50 (42.0)	9 (42.9)	
	Tertiary postgraduate		12 (10.1)	3 (14.3)	
Current smoker			15 (12.6)	2 (9.5)	.46
Preoperative medical history					
Depression			17 (14.3)	4 (19.1)	.52
Anxiety			21 (17.6)	3(14.3)	>.99
Hypertension			42 (35.3)	8 (38.1)	.81
Diabetes mellitus			11 (9.2)	0 (0.0)	.37
Chronic pain (NRS ≥1; ≥3 months duration)			29 (24.4)	12 (57.1)	.004*
Preoperative pain, NRS ≥1 in the last 24 hours (BPI question 5, any anatomical site)			35 (29.4)	13 (61.9)	.006*
Preoperative pain, NRS ≥3 in the last 24 hours (BPI question 5, any anatomical site)			15 (12.6)	9 (42.9)	.002*
Preoperative pain, NRS ≥1 in the last 24 hours (BPI question 5, PPBCS site)			19 (16.0)	10 (47.6)	.002*
Preoperative pain, NRS ≥3 in the last 24 hours (BPI question 5, PPBCS site)			8 (6.7)	7 (33.3)	.002*
Preoperative neuropathic pain (BPI question 5 NRS ≥ 1 & DN4 interview ≥ 3, PPBCS site)			4 (3.4)	4 (19.1)	.02*
Neoadjuvant chemotherapy			3 (2.5)	0 (0.0)	>.99
Surgical and anesthetic					
Surgery type	Breast conserving		84 (70.6)	14 (66.7)	.80
	Mastectomy		35 (29.4)	7 (33.3)	
Axillary surgery	None		9 (7.5)	1 (4.8)	.47
	SNB		93 (78.2)	16 (76.2)	
	AND		17 (14.2)	4 (19.0)	
Reconstruction			15 (12.6)	2 (9.5)	>.99
Repeat surgery			22 (18.5)	7 (33.3)	.15
Anaesthetic modality	Volatile anesthetic		55 (46.2)	15 (71.4)	.06
	TIVA		64 (53.8)	6 (28.6)	
Adjuvant chemotherapy			22 (18.5)	7 (33.3)	.15
Adjuvant radiotherapy			84 (70.6)	17 (81.0)	.43
Hormone therapy			100 (84.0)	18 (85.7)	>.99

Values are presented as n (%) unless indicated. Distribution between categories (no to mild pain and moderate to severe pain) were compared by student t-test for parametric data, or Fisher’s Exact test for assessment of proportions. Mastectomy: simple/total mastectomy, radical/modified radical mastectomy and skin/nipple sparing mastectomy. Breast conserving surgery: excision biopsy, lumpectomy, wide local excision, partial mastectomy, sector resection or quadrantectomy. Repeat surgery: re-resection, breast conserving surgery converted to mastectomy or 2-step Axillary lymph node dissection. Reconstruction surgery: Primary implant prosthesis, autologous flap reconstruction at the time of primary breast surgery.

NRS = numerical rating scale; BPI = short form brief pain inventory; DN4-interview = Douleur Neuropathique en 4 interview; SNB = Sentinel node biopsy; AND = Axillary node dissection; BMI = body mass index; TIVA = Total intravenous anesthesia; SD = Standard Deviation.

**Table 3.** Comparison of pain interference (BPI), upper limb dysfunction (DASH), and psychological distress (DASS21) at 6 months after surgery between patients with no to mild PPBCS versus moderate to severe PPBCS.

	Variable		No to Mild PPBCS (n = 119)	Moderate to Severe PPBCS (n = 21)	*P* value
BPI	Worst pain in past 24 h		0.0 (0.0–1.0)	4 (4.0–6.0)	<.001
	Average pain in past 24 h		0.0 (0.0–1.0)	4 (3.0–5.0)	<.001
	Location of pain	Breast/Chest wall	32 (27.0%)	17 (81.0%)	
		Axilla	28 (23.5%)	15 (71.4%)	
		Shoulder	9 (7.6%)	10 (47.6%)	
		Multiple sites	27 (22.7%)	15 (71.4%)	
	Treatment	Overall analgesic use	11 (9.2%)	8 (38.1%)	
		Paracetamol	9 (2.6%)	8 (38.1%)	
		NSAID	2 (1.7%)	2 (9.5%)	
		Opioid	1 (0.8%)	1 (4.8%)	
		Neuropathic pain agent	2 (1.7%)	3 (14.3%)	
		Multimodal	2 (1.7%)	3 (14.3%)	
	Pain interference (Question 9 average score)		0.0 (0.0–0.3)	2.6 (0.5–3.7)	<.001
		General activity	0.0 (0.0–0.0)	2.0 (0.0–4.0)	<.001
		Mood	0.0 (0.0–0.0)	2.0 (0.0–5.0)	<.001
		Walking activity	0.0 (0.0–0.0)	0.0 (0.0–3.0)	<.001
		Normal work	0.0 (0.0–0.0)	3.0 (0.0–5.0)	<.001
		Relationships with others	0.0 (0.0–0.0)	0.0 (0.0–1.5)	.001
		Sleep	0.0 (0.0–0.0)	4.0 (0.5–5.0)	<.001
		Enjoyment of life	0.0 (0.0–0.0)	3.0 (0.5–5.0)	<.001
DN4 interview	Likely neuropathic pain (DN4 ≥ 3)		20 (16.8%)	9 (42.9%)	.02
DASH	Total DASH score (0–100)		3.3 (0.0–12.5)	20.0 (6.7–38.8)	<.001
DASS21	Depression		0.0 (0.0–1.0)	1.0 (1.0–4.0)	<.001
	Anxiety		0.0 (0.0–1.0)	3.0 (1.0–5.0)	<.001
	Stress		1.0 (0.0–5.0)	6.0 (1.0–8.0)	.004
	Total score		2.0 (0.0–8.0)	9.0 (3.5–18.0)	<.001

Pain interference was derived from the Short form Brief Pain Inventory (BPI) total pain interference score. Total Disability of the Arm Shoulder and Hand (DASH) scores were used to estimate upper limb dysfunction. Douleur Neuropathique en 4 interview (DN4-interview) screened for likely neuropathic pain. Psychological distress was measured as a total Depression, Anxiety, and Stress Scale 21 (DASS21_ score and Depression, Anxiety and Stress sub-item scores. Median values with interquartile range in parenthesis are presented due to non-parametric distribution of the data. Categorical data is presented as n (%). Mann-Whitney *U* test was used to compare groups and Fisher exact test for assessment of proportions.

### Pain development from before surgery to 6 months after surgery

Before surgery, 24 (17.1%) patients reported moderate to severe pain daily in any anatomical location. Of these, 15 (62.5%) patients reported pain in the breast, axilla, shoulder, or arm. Of the 24 patients, 12 (50%) developed any PPBCS (NRS ≥1) at these sites ≥ 6 months after surgery, while 9 (37.5%) developed moderate to severe PPBCS (NRS ≥ 3/10) at these sites ≥ 6 months after surgery. At 2 weeks after surgery, 30.7% (n = 43) patients reported moderate to severe pain at these sites. Of these 43 patients, 29 (67.4) developed any PPBCS (NRS ≥1) at these sites ≥ 6 months after surgery, while 11 (25.6%) developed moderate to severe PPBCS (NRS ≥ 3/10) at these sites ≥ 6 months after surgery.

Of all patients reporting any pain (NRS ≥1) at these sites before surgery, 27.6% (n = 8/29) patients had likely neuropathic pain before surgery (DN4 ≥ 3/7), this increased to 42.1% (n = 43/102) at 2 weeks after surgery. Of the eight patients with likely neuropathic pain before surgery, seven developed any PPBCS (NRS ≥1) at these sites ≥ 6 months after surgery, with six reporting likely neuropathic pain (DN4 ≥ 3/7) ≥ 6 months after surgery.

### Incidence of PPBCS ≥ 6 months after surgery

Overall, 38.5% (n = 54, 95% CI: 30.5%–47.2%) patients reported any PPBCS (NRS ≥1) at these sites ≥ 6 months after surgery. Moderate to severe PPBCS (NRS ≥3) at these sites was reported by 15.0% patients (n = 21, 95% CI: 9.5% - 22.0%) ≥ 6 months after surgery, with a median average BPI pain of 4/10 (IQR: 3–5).

Of the 21 patients who reported moderate to severe PPBCS, 13 (61.9%) reported any pain (NRS ≥1) at any anatomical location before surgery, while 9 (42.9%) reported moderate to severe pain (NRS ≥3) at any site. Of the 21 patients who reported moderate to severe PPBCS, approximately 19 (90.5%) reported any pain at these sites at 2 weeks after surgery, while 12 (57.1%) reported moderate to severe pain.

#### Neuropathic pain

Overall, 40.7% (n = 22/54) of patients with any PPBCS (NRS ≥1) at these sites ≥ 6 months after surgery reported likely neuropathic pain (DN4 ≥ 3/7). Similarly, likely neuropathic pain was found at a significantly higher proportion in patients who reported moderate to severe PPBCS (42.9%, n = 9) compared to those with no to mild PPBCS (16.8%, n = 20) ≥ 6 months after surgery (*P* = .02).

#### Location of PPBCS

Moderate to severe PPBCS was most commonly reported in the breast/chest wall (81.0%) and axilla (71.4%), followed by shoulder (48%). Many patients reported pain at multiple sites (71.4%) (Table 3).

#### Quality of PPBCS

Among patients with moderate to severe PPBCS (NRS ≥3) at these sites ≥ 6 months after surgery (n = 54), the pain was most frequently described as “aching” (85.7%), “tender” (71.4%), “shooting” (71.4%), “sharp” (66.7%), “stabbing” (61.9%), and “tiring-exhausting” (61.9%) on the SF-MPQ-2.

#### Impact of PPBCS

Pain interference, upper limb disability and psychological distress were significantly greater (all *P* < .001) in patients who reported moderate to severe PPBCS at these sites ≥ 6 months after surgery, compared to those with no-mild PPBCS (Table 3).

In patients with moderate to severe PPBCS (≥3/10) at these sites ≥ 6 months after surgery, 38.1% reported using analgesic medications. Few patients in this group (14.3%) reported using multiple analgesics to treat pain with the majority using paracetamol (38.1%), followed by neuropathic pain agents (14.3%), opioids (9.5%), and NSAIDs (9.5%) (Table 3).

### Quantitative sensory testing

There was no significant association (*P* > .37) between preoperative QST (TS, PP40, or CPM) and moderate to severe PPBCS development ≥ 6 months after surgery (Table 4).

**Table 4.** Significant associations between moderate to severe PPBCS with *COMT* rs6269 or rs4818 genotypes and quantitative sensory testing modalities.

Gene	SNP	Model	Genotype	No to Mild PPBCS (n = 119)	Moderate to Severe PPBCS (n = 21)	*P* value
*COMT*	rs6269	Additive	AA	51 (42.8)	5 (23.8)	.01
			GA	51 (42.8)	16 (76.2)	
			GG	17 (14.2)	0 (0.0)	
		Overdominant	AA + GG	68 (57.1)	5 (23.8)	.01
			GA	51 (42.8)	16 (76.2)	
	rs4818	Additive	CC	51 (42.8)	5 (23.8)	.02
			GC	52 (43.7)	16 (76.2)	
			GG	16 (13.4)	0 (0.0)	
		Overdominant	CC + GG	67 (56.3)	5 (23.8)	.01
			GC	52 (43.7)	16 (76.2)	
Preoperative QST	Modality					
	TS			1.3 (0.0–6.0)	1.3 (0.2–8.5)	.64
	PP40 (kPa)			350.3 (252.0–484.3)	330.0 (244.5–431.9)	.61
	CPM (%)			−10.8 (20.5)	−15.1 (17.2)	.37

SNP data are presented as n (%). Quantitative sensory testing (QST) data are presented as mean (standard deviation) or median (interquartile range). Nonparametric data were compared using the Mann-Whitney *U* test and parametric data were compared using the unpaired t-test. Proportions were compared using the χ^2^ test or Fisher exact test. SNP = Single nucleotide polymorphism; TS = temporal summation; PP40 = pressure pain 40; CPM = conditioned pain modulation.

### Genetic factors

The frequencies of SNP for *COMT*, *GCH1*, *ESR1*, *KCNJ6,* and *OPRM1* did not deviate (all *P* > .139) from HWE (Supplementary Table S2). The genotype frequencies for the following SNP pairs: rs6269-rs4818 and rs4633-rs4680, were identical and demonstrated complete linkage disequilibrium (LD) between these SNP pairs (D′=1.00). Characteristics of the genotyped SNPs and the frequencies of *COMT* haplotypes are provided in the Supplementary Tables S2 and S3.

#### Gene associations with PPBCS

Due to the low frequency of the homozygous variant genotype, SNP for *GCH1* (rs8007267, rs1048363), *KCNJ6* (rs2835859) and *OPRM1* (rs1799971, rs563649) could not be assessed using the additive model.

Statistically significant associations between PPBCS and *COMT* (rs6269 and rs4818) were found using both the additive (rs6269 *P* = .01; rs4818 *P* = .01) and overdominant genetic models (rs6269 *P* < .01; rs4818 *P* < .01), (Table 4). There were no other statistically significant associations between other SNP or haplotypes assessed and PPBCS (all *P* > .05).

### Predictors of PPBCS

After correcting for multiple testing, eight variables remained statistically significant (multi-test adjusted *P* < .05) on univariate analysis (table 5). These variables and those having previous associations with PPBCS were selected for multiple logistic regression analysis. Due to the complete LD between *COMT* rs6269 and rs4818, only rs6269 was selected for inclusion in the final model (Table 6).

**Table 5.** Univariate logistic regression analysis for associations of patient, demographic, and treatment factors with moderate to severe PPBCS ≥ 6 months after surgery.

Factor	Category	n (%)	Adjusted Odds Ratio	95% Confidence Interval		*P* value	Multitest Adjusted *P* value
				Lower	Upper		
Age (years)	<50	39 (27.8)	1.00	—	—	—	—
	50–65	55 (39.3)	0.94	0.30	2.95	.91	.95
	>65	46 (32.8)	0.99	0.30	3.23	.98	.99
Ethnicity	Non-European	28 (20.0)	1.00	—	—	—	—
	European	112 (80.0)	0.77	0.25	2.31	.64	.83
Smoker	Never	76 (54.3)	1.00	—	—	—	—
	Current	17 (12.1)	0.59	0.12	2.88	.52	.73
	Ex	47 (33.5)	0.53	0.18	1.57	.25	.49
BMI (kg/m^2^)	<25	40 (28.5)	1.00				
	25–30	52 (37.1)	0.86	0.28	2.60	.79	.89
	>30	48 (34.3)	0.67	0.21	2.20	.51	.73
BPI average preoperative pain at any site	No, NRS = 0	92 (65.7)	1.00	—	—	—	—
	Any, NRS ≥ 1	48 (34.3)	3.90	1.49	10.24	.01	.04
BPI average preoperative pain at any site	No to mild, NRS ≤2	116 (82.9)	1.00	—	—	—	—
	Moderate to Severe NRS ≥ 3	24 (17.1)	5.20	1.88	14.42	.002	.04
Preoperative DASS21	Total score		1.06	1.00	1.12	.04	.14
	Depression sub score		1.18	0.99	1.41	.06	.16
	Anxiety sub score		1.14	0.94	1.38	.17	.38
	Stress sub score		1.11	1.00	1.24	.05	.15
Preoperative QST	CPM		0.99	0.97	1.01	.37	.60
	TS		1.02	0.97	1.08	.36	.60
	PP40		1.00	1.00	1.00	.69	.83
Breast surgery	Breast conserving	98 (70.0)	1.00	—	—	—	—
	Mastectomy	42 (30.0)	1.20	0.45	3.23	.72	.84
Axillary surgery	No	10 (7.1)	1.00				
	SNB	109 (77.8)	1.55	0.18	13.07	.69	.83
	AND	21 (15.0)	2.12	0.20	21.89	.53	.73
ICBN handled or transected	No	78 (55.7)	1.00	—	—	—	—
	Yes	62 (44.2)	0.93	0.37	2.38	.89	.95
ICBN transected	No	103 (73.6)	1.00	—	—	—	
	Yes	37 (26.4)	1.91	0.72	5.06	.19	.40
Reconstruction surgery	No	123 (87.8)	1.00	—	—	—	—
	Yes	17 (12.1)	0.73	0.15	3.45	.69	.83
Anesthetic modality	Volatile	70 (50.0)	1.00	—	—	—	—
	TIVA	70 (50.0)	0.34	0.12	0.95	.04	.14
BPI average pain 2 weeks after surgery at PPBCS site	No, NRS =0	37 (26.4)	1.00	—	—	—	—
	Any, NRS ≥ 1	102 (72.9)	8.24	1.06	63.92	.04	.14
BPI average pain 2 weeks after surgery at PPBCS site	No to mild, NRS ≤2	96 (68.5)	1.00	—	—	—	—
	Moderate to severe, NRS ≥ 3	43 (30.7)	4.26	1.59	11.39	.004	.04
Likely neuropathic pain 2 weeks after surgery (NRS ≥ 1/10, DN4 ≥ 3/7)	No	91 (65.0)	1.00	—	—	—	—
	Yes	48 (34.3)	1.68	0.64	4.39	.29	.54
DASS21 2 weeks after surgery	Total score		1.08	1.03	1.14	.004	.04
	Depression sub score		1.23	1.07	1.42	.01	.04
	Anxiety sub score		1.28	1.07	1.53	.01	.04
	Stress sub score		1.15	1.03	1.28	.02	.06
Repeat surgery	No	111 (79.3)	1.00	—	—	—	—
	Yes	29 (20.7)	2.20	0.80	6.10	.13	.30
Radiation therapy	No	39 (27.8)	1.00	—	—	—	—
	Yes	101 (72.1)	1.77	0.56	5.64	.33	.59
Chemotherapy	No	70 (50.0)	1.00	—	—	—	—
	Yes	70 (50.0)	0.71	0.28	1.82	.48	.73
Endocrine therapy	No	22 (15.7)	1.00	—	—	—	—
	Yes	118 (84.3)	1.14	0.31	4.25	.85	.93
rs6269, *COMT*	GG+AA	73 (52.1)	1.00	—	—	—	—
	GA	67 (47.8)	4.27	1.47	12.41	.01	.04
rs4818, *COMT*	GG or CC	72 (51.4)	1.00	—	—	—	—
	GC	68 (48.5)	4.12	1.42	11.99	.01	.04
rs858003, *KCNJ6*	AA+GA	74 (52.8)	1.00	—	—	—	—
	GG	66 (47.1)	0.39	0.14	1.08	.07	.18

Adjustment for multiple testing were performed by applying the two-stage step-up method of Benjamini, Krieger and Yekutieli.^31^ NRS, Numerical rating scale (0–10), Mastectomy: simple/total mastectomy, radical/modified radical mastectomy and skin/nipple sparing mastectomy. Breast conserving surgery: excision biopsy, lumpectomy, wide local excision, partial mastectomy, sector resection or quadrantectomy. Repeat surgery: re-excision or completion mastectomy. Reconstruction surgery: Prosthetic implant prosthesis, and autologous flap reconstruction at time of primary breast cancer surgery. SNB = sentinel node biopsy; AND = axillary node dissection. ICBN = Intercostobrachial nerve.

**Table 6.** Multiple logistic regression analysis of predictors for developing moderate to severe PPBCS ≥ 6 months after surgery.

Factor	Category	n (%)	B	S.E.	Wald χ^2^	df	*P* value	OR	95% CI	
									Lower	Upper
BPI average preoperative pain at any site	No to mild, NRS ≤2	116 (82.9)								
	Moderate to severe, NRS ≥ 3	24 (17.1)	1.28	0.59	4.71	1.00	.03	3.60	1.13	11.44
Psychological Distress 2 weeks after surgery	DASS21 Total score		0.08	0.03	5.81	1.00	.02	1.08	1.02	1.16
Anesthetic Modality	Volatile (ref)	70 (50.0)								
	TIVA	70 (50.0)	−1.16	0.59	3.91	1.00	.048	0.31	0.10	0.99
*COMT* rs6269	GG + AA (ref)	73 (52.1)								
	GA	67 (47.9)	1.62	0.62	6.75	1.00	.009	5.03	1.49	17.04
*Constant*			−3.29	0.66	24.63	1.00	.00	0.04		

Test		χ^2^		df		*P* value				

Overall model evaluation		26.393		4		<.001				
Likelihood ratio test										
Goodness of fit test		9.25		8		.32				
Hosmer and Lemeshow										
AIC		83.456								
BIC		98.128								
Sensitivity		0.966								
Specificity		0.350								
Correctly Classified, %		87.8								

Nagelkerke R^2^: 0.308. Odds ratio of emotional distress at 2 weeks is presented for every 1-point change in the total DASS21 score.

NRS = Numerical rating scale (0–10); TIVA = total intravenous anesthesia with propofol infusion, S.E. = standard error; B = beta; OR = odds ratio; CI = confidence interval; df = degrees of freedom; AIC = Akaike information criterion; BIC = Bayesian information criterion.

Identified risk factors for PPBCS include moderate to severe pain (NRS ≥3) at any anatomical site before surgery (OR = 3.60, 95% CI = 1.13–11.44, *P* = .03), *COMT* rs6269 GA genotype (OR = 5.03, 95% CI = 1.49–17.04, *P* = .01) and psychological distress at postoperative day 14 (OR = 1.08, 95% CI = 1.02–1.16, *P* = .02). Propofol total intravenous anesthesia (TIVA) was protective for PPBCS (OR = 0.31, 95%CI = 0.10–0.99, *P* = .048). The overall model fit passed Hosmer and Lemeshow Goodness of Fit test = 9.25, *P* = .32 and the Nagelkerke R^2^ value was 0.308 (Table 6). Variance inflation factors for the risk factors included in the final model ranged between 1.01 and 1.08 when assessed, indicating a low potential for multicollinearity.

## Discussion

To our knowledge this prospective cohort study is one of the first comprehensive evaluations of the incidence, impact, and risk factors for PPBCS, that incorporated QST and an exploratory assessment of several candidate gene variants alongside clinical, demographic and treatment-related variables in the risk factor analysis.

The incidence and impact of PPBCS in this study was similar to previous reports.^3–5^ Approximately 15% of patients reported moderate to severe PPBCS ≥ 6 months after surgery, with 42.9% of these patients having likely neuropathic pain (DN4 ≥ 3/7).

Patients with moderate to severe PPBCS had significantly (statistically and clinically) greater pain interference, psychological distress (in each of the depression, anxiety and stress subscales of the DASS21) and upper limb disability. Sleep disruption was the most reported item on the pain interference scale of the BPI (Table 3). In this context sleep disturbance may be both a consequence of pain and a driver to prolong postoperative pain. Regardless, upper limb disability, pain and short-term sleep disturbance all contribute negatively to quality of life.^3^

Only 38.1% of patients with moderate to severe PPBCS reported receiving analgesic medications suggesting that patients either did not report their pain or did not seek medical treatment. Most patients received paracetamol as a single agent with only a few receiving multimodal analgesics. Moreover, first-line anti-neuropathic agents such as tricyclic antidepressants or gabapentinoids were rarely utilized, despite the high incidence of postoperative neuropathic pain.

We identified moderate to severe preoperative pain (irrespective of the site of that pain), and psychological distress 2 weeks after surgery as two potentially modifiable risk factors for PPBCS. Propofol total intravenous anesthesia was identified as a protective factor for PPBCS.

Preoperative and early postoperative pain appear to be important for the development of PPBCS.^6^^,^^7^^,^^9^^,^^13^ In this study, moderate to severe preoperative pain at any site was identified as a risk factor for moderate to severe PPBCS in the final model.^8^ Moderate to severe pain at 2 weeks after surgery, however, although univariately associated with PPBCS, was not included in the best fitting and most parsimonious final model.

To further investigate differences in nociceptive system function that may predispose individuals to developing PPBCS, preoperative QST was undertaken. However, similar to previous studies, there were no associations identified between preoperative QST (CPM, TS and PP40) and the development of PPBCS.^11^^,^^13^ Heterogeneity from confounding factors such as age, cognitive and emotional factors, ethnicity, comorbidities, motivation, QST modality, measurement error, and/or changes in nociceptive system function due to the patients' preoperative pain state may obscure any potential relationship between preoperative QST and PPBCS.^15^ Furthermore, any association between preoperative QST and PPSP may be surgical population specific^16^ and thus factors specific to breast cancer treatment such as intraoperative nerve damage, chemotherapy, radiation therapy and/or endocrine therapy and postoperative emotional distress may influence nociceptive pathway function, and obscure any relationship between preoperative QST and PPBCS.

Negative affective constructs such as general psychological distress, anxiety, depression and pain catastrophizing have long been recognized as preoperative risk factors with a high predictive value for persistent postsurgical pain.^36^ In line with this, heightened psychological distress (as measured by the DASS21) 2 weeks after surgery was a significant independent risk factor for PPBCS development in this study. Furthermore, each DASS21 subscale (depression, anxiety, and stress) was associated with PPBCS in the univariate analysis. The mechanisms by which psychological distress influences PPBCS remain unclear. Negative affect may increase hypervigilance and alter pain processing in limbic regions,^37^ modulate descending modulation of nociceptive pathways,^38^ and enhance inflammation,^39^ all of which may contribute to PPBCS development. Perioperative anxiety may also represent an endophenotype of gene variants such as *COMT* rs4680, previously associated with chronic pain, anxiety, and psychological distress.^40^

In this study, an exploratory assessment of targeted genetic factors was included in the risk factor analysis. The univariate analysis identified the heterozygous genotypes of *COMT* rs6269 (GA) and rs4818 (GC) as risk factors for PPBCS. However, only *COMT* rs6269 was included in the final model due to the complete LD between the two alleles. Interestingly, no patients carrying the rs6269 GG genotype reported PPBCS, supporting previous studies suggesting that the rs6269 G allele is protective for persistent postsurgical pain.^41^ It is noted however, that GA genotype was the strongest risk factor for PPBCS in this study. This may be related to the frequency of the GA genotype and possible heterozygote advantage. Given its location in the *COMT* promoter region, the non-coding rs6269 SNP, most likely regulates *COMT* gene expression and subsequently affects both noradrenergic and dopaminergic transmission in the spinal cord and brain stem; both important pathways in the descending inhibition of pain, and implicated in the chronification of pain.^42^^,^^43^ No other associations between PPBCS and genotype or haplotypes of the other candidate genes were identified, which is not surprising as each SNP may only have a small individual effect, possibly influenced also by epistatic interactions^23^ and possible epigenetic regulation.^44^ Future work incorporating gene expression analysis is required to understand this complex clinical pain syndrome. Furthermore, other gene variants such as inflammatory genes should also be assessed to reflect the variety of molecular changes due to surgical incision, pain sensitization and modulation as the genes assessed in this study are principally involved with nervous system function.^45^

Propofol intravenous anesthesia (TIVA) was identified as a potentially protective factor for PPBCS in this study. There is evidence that TIVA may reduce postoperative pain scores and opioid consumption,^46^ although recent meta-analyses have not been confirmatory.^47^ Similarly, the data for PPBCS specifically is unclear, as both halogenated volatile general anesthetic agents^48^ and propofol infusions^49^ can confer either protection for PPBCS or no benefit. However, as this modality is easily modifiable and may confer other benefits, further investigation is warranted.

This study has limitations. First, the sample size is small, and we did not achieve the target sample size of 220 patients as recruitment was limited by the coronavirus disease 2019 (COVID-19) pandemic. This may have affected the precision of estimated PPBCS incidence. Furthermore, it has caused us to limit our risk factor analysis to variables commonly reported in recent meta-analyses (ignoring others eg, number of preoperative breast biopsies^20^) and may have limited our ability to identify small but clinically important associations between some risk factors and PPBCS. Despite this, the incidence and risk factors of PPBCS reported aligns with international data,^10^^,^^35^ and the MAFs for each SNP were similar to those expected for a predominantly European cohort. However, given our limited sample size, the associations between gene variants and PBBCS should be treated as exploratory. Furthermore, the primary outcome was moderate to severe PPBCS which we defined as pain in the ipsilateral breast, chest wall, axilla, shoulder, or arm ≥ 6 months after surgery with an average pain intensity of ≥3/10. This differs from the IASP definition of chronic postsurgical pain after breast surgery which requires pain to be of new onset, or of increased intensity, lasting more than 3 months after breast surgery after all other causes have been excluded.^50^ We feel however that specifying a 6-month duration within the context of PPBCS is more appropriate, as it allows for completion of chemotherapy and radiation therapy both of which have been described as risk factors for PPBCS.^8^

Moreover, there were statistically significant differences between the groups included and excluded from participating in our study. Patients in the inclusion group were approximately 5 years younger than the excluded group. Previous studies have identified younger age as a risk factor for PPBCS. However, these studies tend to categorize younger age in 10 year decrements,^9^ and thus a 5-year difference may not be biologically relevant. Additionally, a higher proportion of patients in the inclusion group received endocrine therapies as part of their breast cancer treatment. The importance of this is also unclear as high-quality evidence from recent meta-analysis reported no association between endocrine therapy and PPBCS.^2^ Finally, this study was conducted as a single-center study, which may limit the generalizability of its findings.

The study also has important strengths. To our knowledge, it is the first to comprehensively evaluate demographic, clinical, neurophysiological, and genetic risk factors for PPBCS with a 6-month follow-up in a New Zealand cohort, which includes Māori and Pacifica peoples.

Persistent pain after breast cancer surgery appears to be an important consequence of breast cancer treatment. Strategies to improve outcomes will require accurate risk stratification and active, personalized management throughout the treatment course. The specific, potentially modifiable factors identified in this study may inform future intervention studies.

## Supplementary Material

pnad065_Supplementary_Data
