# Supplementary material for: The incidence, impact, and risk factors for moderate to severe persistent pain after breast cancer surgery: a prospective cohort study
Source: Pain Med. 2023 May 15;24(9):1023–34. doi: 10.1093/pm/pnad065 (PMC10655209; doi:10.1093/pm/pnad065)
Supplement: pnad065_Supplementary_Data [file pnad065_supplementary_data.zip › Appendix 2.docx]

**Appendix 2. Quantitative sensory testing protocol**

Temporal summation (TS) was calculated as the difference in pain intensity (0 = no pain; 100 = worst pain imaginable) between the 10^th^ stimulus of a 1 Hz train of stimuli and a single stimulus to a 1 cm^2^ area of skin on the volar forearm ipsilateral to the operative side using a 22.5 g Von Frey filament. (1).

Pressure pain 40 (PP40) was measured on the nailbed of the subject's ipsilateral thumb using a handheld pressure algometer with a probe area of 1 cm and a ramping rate of 10 kPa s^-1^ (Sbmedic Electronics, Solna, Sweden). Patients were instructed to push a button when they experienced pain 40/100 severity (2). PP40 was obtained as the average of three pressure measures at baseline (T1), with a 1-minute rest between measures.

To assess conditioned pain modulation (CPM), the patient's contralateral foot was immersed in cold-water (0-3°C) for a maximum of 2 minutes or until pain level of >4/10 (conditioned stimulus). This was considered sufficient to induce a saturated CPM effect (3). After removing the foot, PP40 was immediately obtained from the ipsilateral thumb (T2). CPM was the percentage change in PP40 after cold water immersion (T2) compared with PP40 alone (T1) and multiplied by -1 so that negative values indicated inhibition (4).

1. Petersen KK, Arendt-Nielsen L, Simonsen O, Wilder-Smith O, Laursen MB. Presurgical assessment of temporal summation of pain predicts the development of chronic postoperative pain 12 months after total knee replacement. *Pain* 2015;**156**(1)**:** 55-61.

2. Yarnitsky D, Bouhassira D, Drewes AM, Fillingim RB, Granot M, Hansson P, Landau R, Marchand S, Matre D, Nilsen KB, Stubhaug A, Treede RD, Wilder-Smith OH. Recommendations on practice of conditioned pain modulation (CPM) testing. *Eur J Pain* 2015;**19**(6)**:** 805-6.

3. Granot M, Weissman-Fogel I, Crispel Y, Pud D, Granovsky Y, Sprecher E, Yarnitsky D. Determinants of endogenous analgesia magnitude in a diffuse noxious inhibitory control (DNIC) paradigm: do conditioning stimulus painfulness, gender and personality variables matter? *Pain* 2008;**136**(1-2)**:** 142-9.

4. Wilder-Smith OH, Schreyer T, Scheffer GJ, Arendt-Nielsen L. Patients with chronic pain after abdominal surgery show less preoperative endogenous pain inhibition and more postoperative hyperalgesia: a pilot study. *J Pain Palliat Care Pharmacother* 2010;**24**(2)**:** 119-28.
