# Supplementary material for: The incidence, impact, and risk factors for moderate to severe persistent pain after breast cancer surgery: a prospective cohort study
Source: Pain Med. 2023 May 15;24(9):1023–34. doi: 10.1093/pm/pnad065 (PMC10655209; doi:10.1093/pm/pnad065)
Supplement: pnad065_Supplementary_Data [file pnad065_supplementary_data.zip › Supplementary Table S1.docx]

**Supplementary Table S1. Demographic, patient, and treatment characteristics of the entire population (n=173) that were recruited to the study.**

| Demographic and Patient Characteristics | | | Total included  (n= 140) | Total excluded (n= 33) | p value |
| --- | --- | --- | --- | --- | --- |
| Mean age (SD) at primary breast surgery; y | | | 57.8 (11.62) | 62.5 (13.49) | 0.05 |
| Mean BMI (SD); kg/m^2^ | | | 28.64 (6.13) | 29.83 (5.82) | 0.31 |
| Ethnicity | European | | 112 (80.0) | 28 (84.8) | 0.63 |
|  | Non-European | | 28 (20.0) | 5 (15.2) |  |
|  |  | Māori | 11 (7.8) | 2 (6.1) |  |
|  |  | Pacific Island | 6 (4.3) | 1 (3.0) |  |
|  |  | Asian | 10 (7.1) | 2 (6.1) |  |
|  |  | Other | 1 (0.7) | 0 (0.0) |  |
| Living with Partner | Yes | | 92 (65.7) | 19 (57.6) | 0.42 |
|  | No | | 48 (34.3) | 14 (42.4) |  |
| Highest Education | Primary/Secondary | | 64 (45.7) | 20 (60.6) | 0.14 |
|  | Tertiary Undergraduate | | 60 (42.8) | 8 (24.2) |  |
|  | Tertiary Postgraduate | | 16 (11.4) | 5 (15.2) |  |
| Depression | Yes |  | 21 (15.0) | 5 (15.2) | >0.95 |
|  | No |  | 119 (85.0) | 28 (84.8) |  |
| Anxiety | Yes |  | 24 (17.1) | 6 (18.2) | >0.95 |
|  | No |  | 116 (82.8) | 27 (81.8) |  |
| Hypertension | Yes |  | 50 (35.7) | 11 (33.3) | 0.84 |
|  | No |  | 90 (64.3) | 22 (66.7) |  |
| Diabetes Mellitus | Yes |  | 11 (7.9) | 2 (6.1) | >0.95 |
|  | No |  | 129 (92.1) | 31 (93.9) |  |
| Chronic Pain | Yes |  | 41 (29.2) | 14 (42.4) | 0.15 |
|  | No |  | 99 (70.7) | 19 (57.5) |  |
| Smoking status | Current |  | 17 (12.1) | 1 (3.0) | 0.20 |
|  | Not current |  | 123 (87.9) | 32 (97.0) |  |
| Surgery Type | Mastectomy |  | 42 (30.0) | 6 (18.2) | 0.20 |
|  | Breast conserving |  | 98 (70.0) | 27 (81.8) |  |
| Axillary Surgery | None |  | 10 (7.1) | 1 (3.0) | 0.68 |
|  | SNB |  | 109 (77.8) | 27 (81.8) |  |
|  | AND |  | 21 (15.0) | 5 (15.2) |  |
| Reconstruction | Yes |  | 14 (10.0) | 2 (6.1) | 0.74 |
|  | No |  | 126 (90.0) | 31 (93.9) |  |
| Repeat Surgery | Yes |  | 29 (20.7) | 7 (21.2) | >0.95 |
|  | No |  | 111 (79.2) | 26 (78.9) |  |
| Adjuvant Chemotherapy | Yes |  | 70 (50.0) | 12 (36.4) | 0.18 |
|  | No |  | 70 (50.0) | 21 (63.6) |  |
| Adjuvant Radiotherapy | Yes |  | 106 (75.7) | 28 (84.8) | 0.36 |
|  | No |  | 34 (24.3) | 5 (15.2) |  |
| Hormone Therapy | Yes |  | 118 (84.3) | 20 (60.6) | 0.01 |
|  | No |  | 22 (15.70) | 13 (39.4) |  |

Values are presented as n (%) unless indicated. Non-parametric data are presented as median with interquartile range in parenthesis. Distribution between included and excluded groups were compared by student t-test for parametric data, or Fisher’s Exact test for assessment of distribution. Mastectomy: simple/total mastectomy, radical /modified radical mastectomy and skin/nipple sparing mastectomy. Breast conserving surgery: excision biopsy, lumpectomy, wide local excision, partial mastectomy, sector resection or quadrantectomy. Repeat surgery: re-resection, breast conserving surgery converted to mastectomy or 2-step Axillary lymph node dissection. Reconstruction surgery: Expander or implant prosthesis, autologous flap reconstruction. SNB, Sentinel node biopsy; AND, Axillary node dissection; BMI, body mass index; SD, Standard Deviation.
