# Supplementary material for: The incidence, impact, and risk factors for moderate to severe persistent pain after breast cancer surgery: a prospective cohort study
Source: Pain Med. 2023 May 15;24(9):1023–34. doi: 10.1093/pm/pnad065 (PMC10655209; doi:10.1093/pm/pnad065)
Supplement: pnad065_Supplementary_Data [file pnad065_supplementary_data.zip › Supplementary Table S3.docx]

**Supplementary Table S3. Frequencies of *COMT* Haplotype (rs6269, rs4633, rs4818, rs4680)**

| Haplotype | Frequency,  n (%) |
| --- | --- |
| GCGG | 100 (35.7) |
| ATCA | 138 (49.3) |
| ACCG | 41 (14.6) |
| GCCG | 1 (0.4) |
