# Supplementary material for: The incidence, impact, and risk factors for moderate to severe persistent pain after breast cancer surgery: a prospective cohort study
Source: Pain Med. 2023 May 15;24(9):1023–34. doi: 10.1093/pm/pnad065 (PMC10655209; doi:10.1093/pm/pnad065)
Supplement: pnad065_Supplementary_Data [file pnad065_supplementary_data.zip › Supplemetary Table S2.docx]

| Gene | SNP ID | Chromosomal Position, GRCh38 38.1/141 | Functional Location | Alleles (major, minor) | Minor Allele Frequency |  | HWE p |
| --- | --- | --- | --- | --- | --- | --- | --- |
| *COMT* | rs6269 | Chr22: 19962429 | Intron variant | A,G | 0.36 |  | 0.66 |
|  | rs4633 | Chr22: 19962712 | Synonymous variant | C,T | 0.49 |  | 0.50 |
|  | rs4818 | Chr22: 19963684 | Synonymous variant | C,G | 0.36 |  | 0.49 |
|  | rs4680 | Chr22: 19963748 | Missense variant | G,A | 0.49 |  | 0.50 |
| *GCH1* | rs8007267 | Chr14: 54912273 | None | C,T | 0.18 |  | 0.38 |
|  | rs3783641 | Chr14: 54893421 | Intron variant | T,A | 0.21 |  | 0.61 |
|  | rs10483639 | Chr14: 54839739 | None | G,C | 0.18 |  | 0.84 |
| *ESR1* | rs3020377 | Chr6: 151951263 | Intron variant | A,G | 0.41 |  | 0.99 |
|  | rs2234693 | Chr6: 151842200 | Intron variant | T,C | 0.44 |  | 0.88 |
|  | rs9340799 | Chr6: 151842246 | Intron variant | A,G | 0.32 |  | 0.47 |
| *KCNJ6* | rs2835925 | Chr21: 37747000 | Intron Variant | A,G | 0.24 |  | 0.65 |
|  | rs858003 | Chr21: 37700681 | Intron Variant | G,A | 0.30 |  | 0.18 |
|  | rs2835859 | Chr21: 37645860 | Intron variant | T,C | 0.09 |  | 0.36 |
| *OPRM1* | rs1799971 | Chr6: 154039662 | Missense variant | A,G | 0.14 |  | 0.14 |
|  | rs563649 | Chr6: 154086832 | Intron variant | C,T | 0.09 |  | 0.29 |

Supplementary Table S2. Characteristics of the genotyped SNPs

HWE: Hardy-Weinberg equilibrium. The genotype frequencies for the SNP pairs: rs6269-rs4818 and rs4633-rs4680, were identical and demonstrated complete LD between these SNP pairs (D’=1.00)
