# Supplementary material for: The incidence, impact, and risk factors for moderate to severe persistent pain after breast cancer surgery: a prospective cohort study
Source: Pain Med. 2023 May 15;24(9):1023–34. doi: 10.1093/pm/pnad065 (PMC10655209; doi:10.1093/pm/pnad065)
Supplement: pnad065_Supplementary_Data [file pnad065_supplementary_data.zip › Appendix 1.docx]

**Appendix 1. Simplified anesthetic protocol.**

The decision of specific anesthetic modality (Inhalational versus total intravenous anesthesia) was left to the attending anesthetist. No routine premedication was administered. General anesthesia was maintained using volatile anesthesia (sevoflurane) or propofol total intravenous anesthesia (TIVA). All patients received dexamethasone (4-8 mg) with or without droperidol (0.625 mg) for postoperative nausea and vomiting prophylaxis. Intraoperative analgesia included parecoxib (40 mg), and opioid analgesia (fentanyl, morphine or oxycodone). Local anesthetic was infiltrated into the wound by the surgeon before closure according to the surgeon’s own preference.

Postoperatively, patients received paracetamol, NSAID (ibuprofen, diclofenac or celecoxib), tramadol, and an antiemetic protocol (dexamethasone, ondansetron, cyclizine and/or droperidol) as required. Patients with poorly controlled pain received intravenous/oral opioids (fentanyl, morphine or oxycodone) for rescue analgesia at the discretion of the attending anesthesiologist. Perioperative ketamine or regional anesthesia were not permitted. Reasons for deviations from this protocol were recorded.
